# EEG Decoding Reveals the Strength and Temporal Dynamics of Goal-Relevant Representations

**DOI:** 10.1038/s41598-019-45333-6

**Published:** 2019-06-21

**Authors:** Jason Hubbard, Atsushi Kikumoto, Ulrich Mayr

**Affiliations:** University of Oregon, Eugene, OR 97403 United States

**Keywords:** Cognitive control, Attention

## Abstract

Models of action control assume that attentional control settings regulate the processing of lower-level stimulus/response representations. Yet, little is known about how exactly control and sensory/response representations relate to each other to produce goal-directed behavior. Addressing this question requires time-resolved information about the strength of the different, potentially overlapping representations, on a trial-by-trial basis. Using a cued task-switching paradigm, we show that information about relevant representations can be extracted through decoding analyses from the scalp electrophysiological signal (EEG) with high temporal resolution. Peaks in representational strength—indexed through decoding accuracy—proceeded from superficial task cues, to stimulus locations, to features/responses. In addition, attentional-set representations were prominent throughout almost the entire processing cascade. Trial-by-trial analyses provided detailed information about when and to what degree different representations predict performance, with attentional settings emerging as a strong and consistent predictor of within-individual and across-individual variability in performance. Also, the strength of attentional sets was related to target representations early in the post-stimulus period and to feature/response representations at a later period, suggesting control of successive, lower-level representations in a concurrent manner. These results demonstrate a powerful approach towards uncovering different stages of information processing and their relative importance for performance.

## Introduction

**Figure 1:**
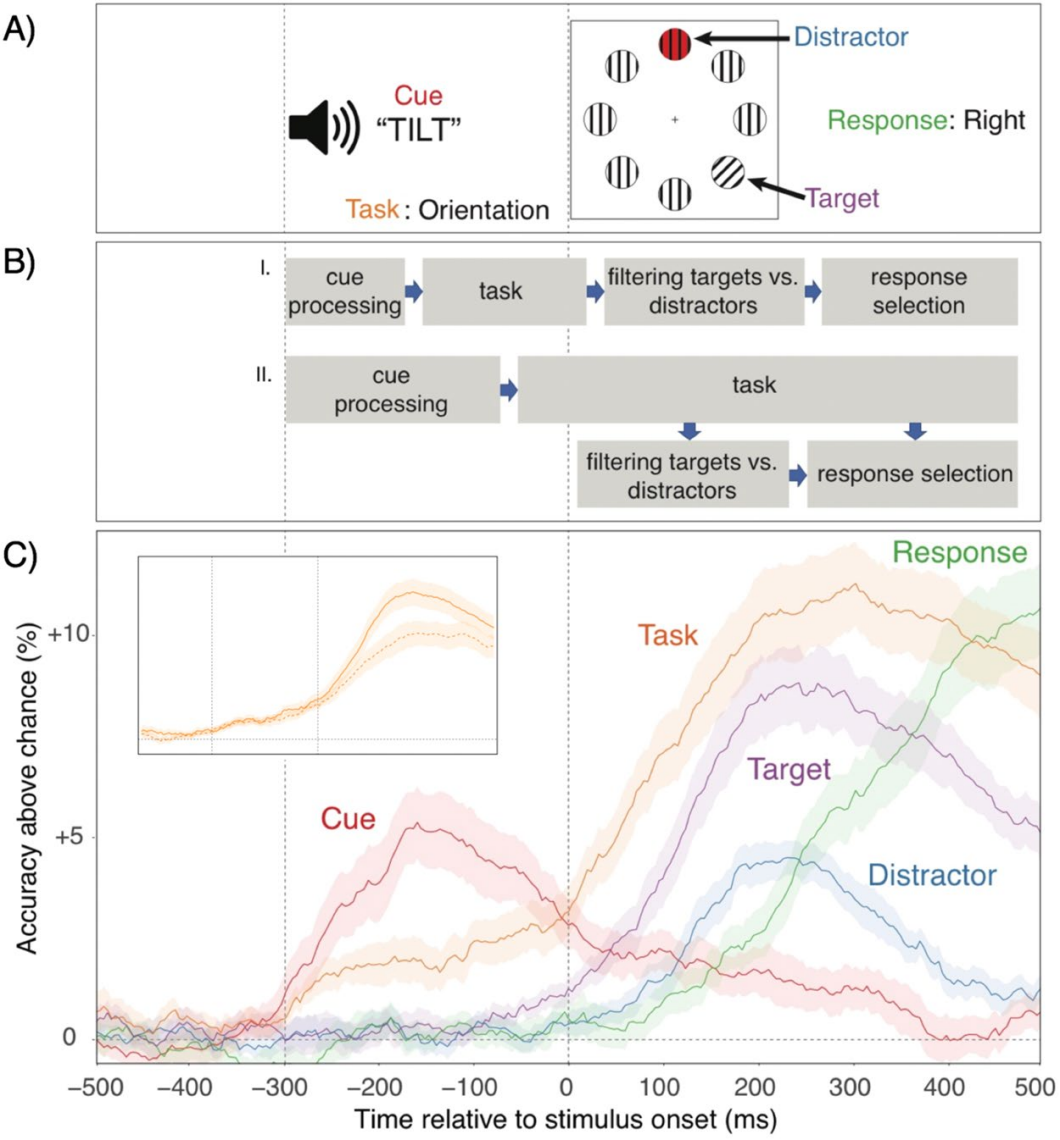
(**A**) Stimulus timeline with each relevant task aspect. (**B**) Competing models specifying either a (I) preconfiguration or a (II) parallel-activation relationship between attentional set and stimulus/response representations. (**C**) Decoding accuracy of each aspect across time, relative to chance (*p* = 0.5, except for target and distractor, where it was *p* = 0.25). In all figures, shaded regions specify 95% within-subject confidence intervals. The insert shows how task (i.e., attentional-set) decoding accuracy generalizes both within (filled line) and across target locations/responses (dotted line). Note, that in the current work we are particularly interested in within-individual variability in decoding accuracy and therefore the values presented here are based on averaged, trial-by-trial results. When performing decoding analyses based on averaged data, much higher decoding accuracy (>80% for some aspects) can be achieved.

The efficiency of information processing differs both moment to moment, and from one individual to the next. Such variability could reflect the quality of low-level, stimulus or response representations. Alternatively, it may arise from the strength of more abstract, attentional settings that instantiate or control sensory and response-related processes^1–3^. For example, in the experimental paradigm we used in the current work (see Fig. 1A), participants were informed on each trial through auditory cues, which of two attentional settings to use^4,5^. For the Color task, they attended to the color singleton within the array of objects and responded via button press whether the exact color was orange or purple. Similarly, for the Orientation task, participants attended the orientation singleton and responded whether the line tilted to the left or to the right. In this situation, successful performance requires lower-level representations of the task cue, of the target location, and of the task-relevant feature/response. However, it may also require abstract attentional-set representations that differentiate between the color task context and the orientation task context and that ensure an adequate cascade of lower-level representations.

Even though the existence of higher-level, task or rule representations is a common assumption in models of cognitive control^3,6–8^ there are open questions about the degree to which such more abstract representations regulate performance, and how such regulation is achieved^4,5,9,10^. For example, as an alternative to the view that abstract, task-level representations are necessary to modulate lower-level processes, some authors have pointed out that when unambiguous, environmental stimuli (i.e., cues) distinguish between competing response options, superficial cue representations could be sufficient to constrain lower-level processes^4^. It is also currently not clear when exactly higher-level control occurs (see Fig. 1B). Cue or task-set representations might be necessary to set up and preconfigure lower-level representations^11,12^. Alternatively, task sets may also become relevant only as competition between lower-level representations arises, in order to mold these representations in a goal-appropriate manner ^3^. Addressing these and related issues requires methods that directly probe the status and functional relevance of goal-relevant representations with high temporal resolution.

Existing approaches, such as chronometric analyses of response-time (RT) patterns^13^, the analysis of averaged evoked EEG^14^, or fMRI BOLD signals^15^ are of limited value for capturing temporal dynamics, or trial-to-trial variability (but see^16^, in the strength of different task-related representations. A particularly challenging problem is to identify representations that overlap in time, such as when attentional-set and stimulus/response representations occur and influence each other simultaneously. Moreover, because abstract attentional sets are not tied to specific stimuli or responses, they are particularly difficult to pin down with the existing methods.

The limited knowledge we have about the flow of information processing in humans, stands in contrast to advances from primate neurophysiological research. For example, in a recent study that served as a model for the current work, Siegel and colleagues^17^ had monkeys perform a task-switching paradigm while multi-unit activity was recorded in critical anatomical areas along the entire sensory-motor processing stream^18–20^. The results suggest that abstract task rules, rather than superficial cue representations, control lower-level processes in a largely parallel manner. Specifically, the neural coding of cue information was very robust during the pre-stimulus phase, but then tampered off in the post-stimulus phase. In contrast, task-level information emerged concurrently with cue information, but then increased dramatically as stimulus and response choice information was processed during the entire response phase.

In humans it is currently not possible to match this type of primate, neurophysiological research in terms of both temporal and neuroanatomical resolution. However, recent work has suggested that a surprising amount of information about currently active representations can be extracted from EEG signals^21–25^. This approach is similar to the multi-voxel-pattern-classification method in fMRI research, where the pattern of activations across a set of voxels is used to classify specific task aspects (e.g., face vs. house stimuli). The degree to which such classification is possible, indicates the strength with which that aspect is encoded within the neural signal. Applied to EEG, the spatial pattern of the power in specific frequency bands (or of the raw EEG signal) across electrodes is used to categorize the task aspect in question. Recent work has shown that among other aspects, the focus of spatial attention^23^, the content of spatial working memory^22^, and even of semantic categories^24^ can be decoded from the EEG signal. While EEG-based decoding results are limited in their spatial resolution (compared to fMRI), the decoding analyses can be repeated for every time point, thus yielding very high temporal resolution.

We applied the EEG decoding approach to the task-switching paradigm presented in Fig. 1A. Our goal was to track the temporal dynamics of activating the five, potentially relevant aspects: task cues, target and distractor locations, target feature/response, and the attentional/task set. In addition, we conducted our decoding analyses on the single-trial level, which provided information about a particular aspect’s decoding strength for each time point and each trial. This allowed us to examine with high temporal resolution the relative importance of lower-level, stimulus/response, versus higher-level attentional-set representations in predicting trial-by-trial variability in performance.

## Methods

### Participants

Consistent with the sample sizes used in other recent EEG-decoding experiments^22–24^, a total of 22 participants participated in this experiment. One participant was excluded for having EEG artifacts in excess of 30% of trials, and one was excluded due to an experimenter error that resulted in data loss, leaving a sample size of *N* = 20. Participants were compensated at a rate of $10 per hour, with additional incentives based on performance on the task. All experimental procedures were approved through the University of Oregon’s Human Subject Review Board, and performed in accordance with relevant guidelines/regulations. Informed consent was obtained from all participants.

### Tasks and stimuli

We used a cued task switching paradigm that was closely modelled after a paradigm that we had previously used in the context of eye-tracking experiments^12,26^. On each trial, an auditory cue indicated which of the tasks, the Color task or the Orientation task, participants had to complete. Each task was paired with two auditory cues: “color” or “hue” for the Color task, and “tilt” or “lean” for the Orientation task. We used two sets of cues (Set A = “color” and “tilt”, Set B = “hue” and “lean”) that were alternated across consecutive trials^27^. Because with this procedure, both task-repeat and task-switch transitions were accompanied by cue transitions, it ensured that task-switch costs are not contaminated by superficial cue-priming effects^4,5^ and that we could independently decode cue and task information^17^.

The stimulus array consisted of 8 circular gratings (diameter of each ~2.4 degrees) in a larger circular arrangement (diameter ~12.5 degrees). The stimulus array always contained six, neutral stimuli consisting of vertical, black and white gratings. In addition, there was (a) one color singleton stimulus with a vertical grating shaded in one of two colors, either “yellowish” or “reddish” and one (b) orientation singleton with a black grating oriented either 30 degrees to the left or the right. For the Color task, participants had to attend to the color singleton and press the left (z) key for a “yellowish” target and the right (/) key for the “reddish” target. For the Orientation task, participants had to attend to the orientation singleton and press the left (z) key for a left-tilted target and the right (/) key for the right-tilted target.

Each trial began with a 700 ms prestimulus interval with a fixation cross in the center of the screen. The auditory cue was presented in the last 300 ms of this interval, so that the stimulus array appeared as soon as the auditory cue completed. Participants were instructed to respond as quickly and accurately as possible. The stimuli remained on the screen until a response was made. In case of a mistake, an error tone was emitted for 100 ms. During the following inter-trial interval (ITI), which was jittered between 750 and 937 ms, participants were instructed to blink before the next trial began.

The experiment began with two single-task practice blocks (one for each task, order counter-balanced), and a task-switching practice block (20 trials), followed by 22 test blocks of 64 trials each. In order to incentivize participants to respond quickly and accurately, they were rewarded a small amount (0.5 cents) for each trial where they were faster than the 75^th^% percentile of their RT distribution up to that point, but only if they maintained at least 90% accuracy for a given block^26^. At the end of each block, subjects were given feedback about their average RT and accuracy for that block. The RT distribution was determined separately for each task and switch condition after the first mixed-task block, and updated with each trial. All task aspects were determined randomly on a trial-by-trial basis. This includes the selection of tasks, yielding an average switch rate of *p* = 0.5.

Participants were seated approximately 70 cm from the screen, and instructed to keep their eyes at fixation and not blink throughout the trial.

### EEG Recording and Preprocessing

Electroencephalographic (EEG) activity was recorded from 20 tin electrodes held in place by an elastic cap (Electrocap International) using the International 10/20 system. The 10/20 sites F3, Fz, F4, T3, C3, CZ, C4, T4, P3, PZ, P4, T5, T6, O1, and O2 were used along with five nonstandard sites: OL midway between T5 and O1; OR midway between T6 and O2; PO3 midway between P3 and OL; PO4 midway between P4 and OR; and POz midway between PO3 and PO4. The left-mastoid was used as reference for all recording sites. Data were re-referenced off-line to the average of all scalp electrodes. Electrodes placed ~1 cm to the left and right of the external canthi of each eye recorded horizontal electrooculogram (EOG) to measure horizontal saccades. To detect blinks, vertical EOG was recorded from an electrode placed beneath the left eye and reference to the left mastoid. The EEG and EOG were amplified with an SA Instrumentation amplifier with a bandpass filter of 0.01–80 Hz and were digitized at 250 Hz in LabView 6.1 running on a PC. Preprocessing was performed using the Signal Processing and EEGLAB^28^ toolboxes in MATLAB. Trials including blinks (>80uv, window size = 200 ms, window step = 50 ms), large eye movements (>1°, window size = 200 ms, window step = 10 ms), and blocking of signals (range = −0.0 5uv to 0.05uv, window size = 200 ms) within the interval of −700 to +400 ms relative to the stimulus were rejected and excluded from further analysis, resulting in an average of 180 trials (12.4%) rejected across participants.

After the initial preprocessing, the single-trial EEG data were decomposed into a time-frequency representation via wavelet decomposition. The power spectrum of the EEG signal was obtained through a fast Fourier transform, which was then convolved with the power spectrum of complex Morlet wavelets, defined by (${e}^{2\pi ft}{e}^{-t2/(2\ast {\sigma }^{2})}$), where *t* is time, *f* is frequency, and σ is the width of each frequency band, set according to *n*/*2πf*, with *n* increasing logarithmically from 3 to 8. This was repeated for the frequency bands between 2 and 31 Hz in logarithmically-spaced steps. The incremental number of wavelet cycles was used to balance between both temporally-based and frequency-based precision^29^. The results were then brought back into the temporal domain using an inverse Fourier transform. A frequency band-specific estimate at each time point was defined as the squared magnitude of the convolved signal *Z*(real([*z*(*t*)]^2^ + imag[*z*(*t*)]^2^) for power. Only power was considered for the present investigation, and for simplicity we focused on frequency bands that are most often presented in the literature: delta (2–3 Hz), theta (4–7 Hz), alpha (8–12 Hz), and beta (13–31 Hz). For each frequency band, we averaged the power signal across the range of interest. Note, that for the decoding analyses we did not differentiate between the four frequency bands.

### Decoding analyses

With the decoding analyses, we examined the extent to which the spatial pattern of the EEG power across the scalp was predictive of each task aspect. The aspects we considered were the auditory cue (“color”/“hue” or “tilt”/“lean”, classified within tasks), the task (Color or Orientation), the target position (partitioned into 4 bins, coded 1–4), the distractor position (bins 1–4), and the response (left vs. right).

For the target/distractor location, we decoded positions based on the bin that each item appeared in (e.g., bin 1 = top and top-right position, bin 2 = right and bottom-right position, etc.). This ensured that the target and distractor occupied each combination of bins with equal frequency (including sharing the same bin), thus ensuring that successful distractor decoding is not simply due to the classifier decoding “not target position”.

Note also that in the current paradigm we cannot distinguish between the manual response and the specific target stimulus (e.g., left-tilted grating, or reddish grating) as they were confounded. Also, as we wanted to isolate the discriminability of each aspect regardless of any task differences, we performed the decoding separately within each task (except, of course in decoding the task set itself). The results from these analyses were then averaged. Previous work has established that different types of information are encoded in brain oscillations at particular frequency bands^22,30,31^, which motivated decomposing the raw EEG signal into the separate bands. However, in the present investigation we were agnostic to which bands encode which type of information, and thus concatenated all 4 bands together in the decoding analysis.

For each trial, we extracted a window centered around stimulus time onset, starting 500 ms before and extending 500 ms after the onset. The end of this interval corresponds to the 70^th^ percentile of the RT distribution, ensuring that at least 70% of trials are still in progress at that point. We performed the analyses separately for each 4 ms time point. Thus, in the decoding analyses, the features consisted of the estimate of power for each electrode at a single point in time, repeated for each frequency band (20 electrodes × 1 time point × 4 bands = 80 features). Prior to decoding, the EEG data were z-scored so that the mean of each trial’s data was 0 without baseline activity subtraction. We performed all analyses separately for each subject and then averaged results across subjects (e.g.^22^). We used L2-regularized logistic regression, as implemented in the scikit-learn package in Python^32^, with a tolerance of 1 × 10^–4^ and the inverse of the regularization strength (C) set to 1.0. Multi-class classification (which was necessary for target and distractor positions), was implemented as a series of binary classifications. For all decoding analyses, we used a 4-fold cross-validation procedure where 75% of trials were used in the training set, and the remaining 25% of trials were used as the test set, and this was repeated until each trial had an opportunity to be part of the test set. We also repeated the analyses with naïve Bayes, support vector machines, and random forests. The results were consistent across the different algorithms.

**Figure 2:**
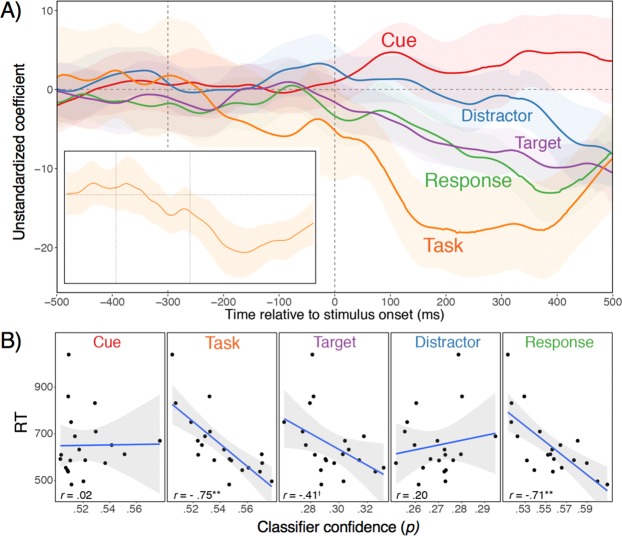
Within and between-individual relationships between decodability of all task aspects and RTs. (**A**) Coefficients from multilevel, linear models with logit-transformed classifier evidence from all task aspects for a given timepoint simultaneously predicting RTs. The insert shows the coefficient when the task-set predictor is based on the generalization scores presented in the insert to Fig. 1C. (**B**) *T*-values representing simple correlations between individuals’ average, logit-transformed classifier evidence for each task aspect and their average RT. (**C**) Scatterplots of relationships between subject-averaged classifier evidence and RTs during peak, average decoding accuracy periods for each task aspect. For task-level generalization scores (see insert to Fig. 1C), the correlation remained very robust at 0.63 (*p* < 0.01).

**Figure 3:**
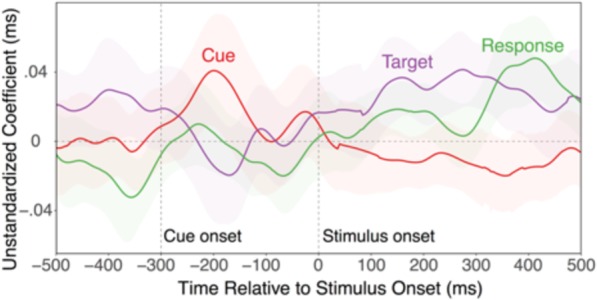
Coefficients representing the independent relationships between classifier confidence for task-level decoding on the one hand, and for cue, target, and responses on the other. For clarity of presentation, the relationship with distractor classifier confidence was omitted here, which hovered around 0 throughout.

**Figure 4:**
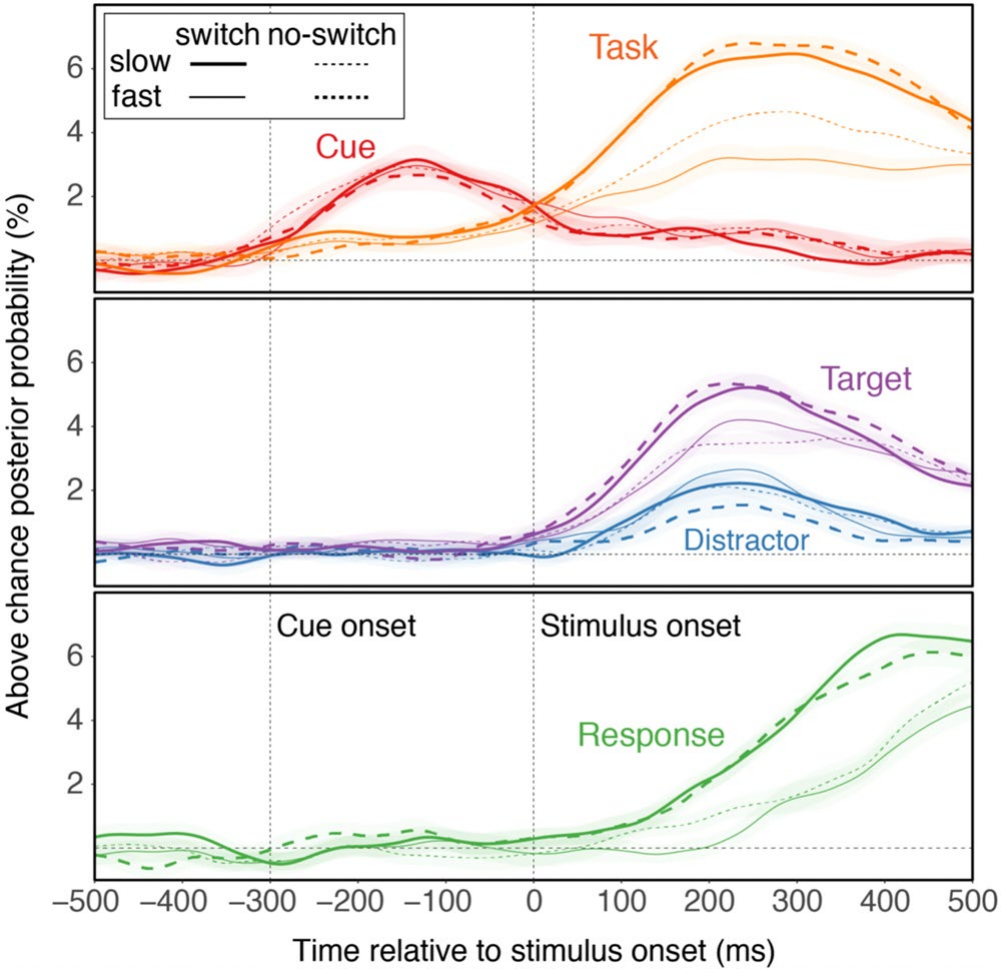
Average classifier confidence, separately for switch vs. no-switch trials and fast vs. slow RTs (determined via median split within individuals, tasks, and switch vs. no-switch trials).

**Figure 5:**
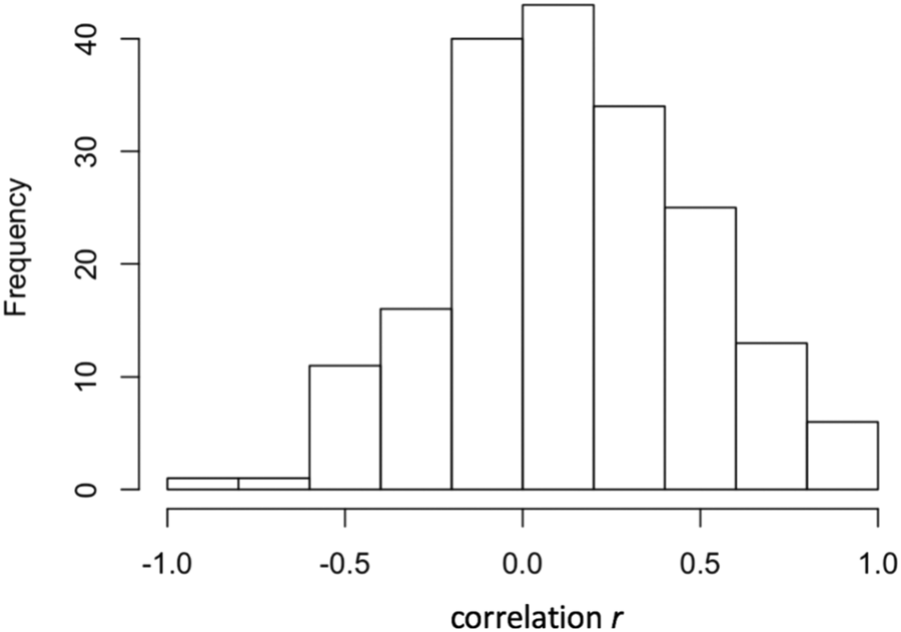
Histogram of correlations of task-contrast t-values across electrodes and frequency bands for all possible pairs of individual subjects (i.e., one correlation per pair).

For the main decoding analysis (Fig. 1C in manuscript), we reported the decoding accuracy, averaged within subject, timepoint, and factors of interest (e.g., task), and then across subjects. We used decoding accuracy here as it is most consistent with how such results are presented in the literature. In contrast, Figs 2–5 are based on classifier confidence, which provide a continuous prediction score on the trial-by-trial level. Specifically, for each item in the test set, the classifier generates a probability (using the predict_proba function in scikit-learn), indicating the classifier’s confidence that the test observation belongs to each class. The class with the highest posterior corresponds to the prediction given by the classifier, and the sum of all probabilities for a single test observation equals 1. Average patterns of results based on classifier confidence are qualitatively very similar to those based on decoding accuracy, but are smoother and with tighter confidence intervals than for average decoding accuracy. Most importantly, the continuous score is better suited for the analyses of trial-to-trial variability as presented in Fig. 2.

For some of the subsequent analyses (e.g., Figs 2C and 5), we derived decoding scores that reflected the peaks of decoding accuracy for each feature. For these analyses, we identified the average time point with the maximum decoding accuracy for each aspect (see Fig. 1C) and averaged the (logit-transformed) classifier confidence across a 150 ms window centered around that point. For the response aspect, the maximum decoding accuracy was towards the end of the 500 ms interval. Here, we simply averaged the period from 350–500 ms.

In the Supplemental Information, we provide additional analyses to (a) rule out the effects of eye-movements on task decoding, (b) examine task-specific effects on the encoding of lower-level features, and (c) provide additional information about trial-by-trial predictive relationships between decoding accuracy and behavior.

## Results

### Behavior

Table 1 contains mean RTs and error rates as a function of task and switch condition. We submitted RTs to a repeated-measures ANOVA with the factors task and switch. This yielded a modest, but highly significant switch effect, *F*(1,19) = 40.7, *p* < 0.001, but no effect of task, *F*(1,19) = 0.03, *p* = 0.86, and only a marginal task × switch interaction, *F*(1,19) = 3.33, *p* = 0.08. For error rates, we obtained a main effect for task, *F*(1,19) = 13.27, *p* = 0.002, with higher error rates for the color than for the orientation task, a main effect for switch, *F*(1,19) = 14.53, *p* = 0.001, and also a task × switch interaction, *F*(1,19) = 10.05, *p* = 0.005, with somewhat larger switch costs for the color task.

**Table 1:** RTs and error rates as a function of task and switch contrast.

	Color Task		Orientation Task	
	no-switch	switch	no-switch	switch
RT (ms)	632 (238)	663 (230)	636 (195)	653 (204)
errors (%)	3.86 (1.80)	5.92 (2.19)	2.23 (1.30)	2.74 (1.45)

The magnitude of RT switch costs was relatively small. This is likely due to two design factors in our paradigm. First, to allow blinks between trials we used a relatively long response-stimulus interval (RSI) that ranged between 1450 and 1637 ms. Second, different from the more standard switching paradigm, where a central stimulus combines all task-relevant aspects, in the current paradigm target and distractors were spatially separated in order to allow decoding of target and distractor locations as task-related aspects. Given the spatial separation between targets and distractors, participants could use spatial attention to reduce or even eliminate stimulus-induced, between-task interference, which is critical for obtaining large task-switch costs^33^.

At least in terms of errors, the orientation task was somewhat easier than the color task, which may seem surprising given that color information is typically more salient than orientation/shape information. Likely this is due to the fact that the left-versus-right orientation discrimination has a compatible stimulus-response mapping (i.e., right tilt- > right response, left tilt- > left response), whereas the response mapping for the color task is arbitrary.

### Representational dynamics

Figure 1C shows that decoding accuracy unfolds in a manner that is consistent with standard expectations about the flow of information––from cue encoding, to attentional-set activation, to relevant and irrelevant stimulus locations, and finally to feature/response codes. Remarkably, task-level (i.e., attentional set) information was decodable with high accuracy throughout almost the entire duration of the trial.

To ensure our conclusions regarding the decoding of the theoretically critical task feature are not compromised by multiple comparisons and faulty comparisons against chance^34,35^, we also conducted a cluster-based permutation test. First, we generated a series of t-values via t-tests against the chance level (33.3%) using decoding results with randomly shuffled labels. Then, we identified reliable clusters as neighboring time points that exceeded a primary threshold (cluster-defining threshold, alpha = 0.05) and retained the size of the maximum cluster. We computed a cluster *p-*value under the permutation distribution of cluster-level statistics (5000 permutations), which defined clusters as significant if their size was larger than the 95th (i.e., alpha = 0.05) largest member in the permutation distribution. According to these analyses, we identified a significant cluster expanding from −260 to 500 ms for the task feature (cluster- defining threshold, *p* < 0.05, corrected significance level, *p* < 0.001, critical statistics = 52 ms).

As mentioned in the Introduction, one prominent model suggests that the cognitive system does not actually rely on abstract task settings, but––at least when available-–uses superficial cue representations to resolve ambiguity between competing stimulus/response representations^4,9^. However, we found that cue decoding accuracy (i.e., discriminating between the two cues for each task) peaks during the pre-stimulus phase, but declines sharply once the stimulus is presented. This result is consistent with the view that cue representations are used to activate task- or attentional-set representations^5,27^ and are less involved with actually regulating task-specific processes.

But how exactly are attentional sets instantiated? If such settings are critical for “preconfiguring” lower-level processes, stimulus representations would need to wait until they are firmly established^11,12^. Alternatively, task sets may be activated in parallel to low-level stimulus/response selection processes, biasing these in a goal-relevant direction^3^. As shown in Fig. 1C, there was above-chance task decoding during the prestimulus phase, which substantially increased once stimulus/response information became decodable. Thus, while the presence of prestimulus, task-level information indicates some role for preconfiguration, the overall pattern suggests that––consistent with the parallel-activation hypothesis––attentional settings become particularly critical once competition between conflicting stimulus/response representations needs to be resolved (see Fig. 1B).

### Determining the relevance of representations

Going beyond average activation trajectories, the trial-by-trial decoding approach allows determining at what point in the trial, which of the represented aspects drive performance. For this purpose, we entered logit-transformed classifier probabilities for the five aspects as simultaneous predictors into a linear mixed-effects model, predicting trial-by-trial RTs, with a random intercept and random coefficients for each subject using the lme4 package^36^. RTs were pre-whitened by removing any linear or quadratic trends across the blocks of the experiment. The coefficients shown in Fig. 2A represent the unique predictive power associated with each aspect, as a function of time in the trial. Note that negative coefficients imply that the greater the classifier confidence, the faster the RTs—indicating that classifier confidence can be interpreted as representational strength. Consistent with the view that attentional sets, and not superficial cues, control lower-level representations, we found that cue-related activity is largely irrelevant for performance, a result that also holds up when cue decoding accuracy is entered as the sole predictor. In contrast, attentional-set information became highly predictive of RTs during the post-stimulus phase, suggesting that fluctuations in the quality of attentional-sets are a major source of trial-to-trial variability in performance. Consistent with the parallel-activation account, attentional sets begin to predict RTs only once also the (independent) predictive power of stimulus and response information emerges. It is noteworthy that the predictive effect for task-level decoding emerges over and above the—also substantial—predictive effects for the target location and the feature/response aspect. Moreover, the aspects that we do not expect to predict performance (i.e., the cue and the distractor location) show no relationships. This result rules out the possibility that fast-RT trials are simply less noisy and therefore allow better decoding of any aspect.

We also looked at the degree to which the decoding accuracy for the different aspects is related to individual differences in RTs. We found that the temporal pattern of simple correlations between individuals’ average decoding accuracy for each task aspect and their average RT was very similar to the within-individual predictive pattern. As shown in Fig. 2B, decoding accuracy for attentional sets was a major source of individual differences in RTs for nearly the entire post-stimulus period, whereas stimulus location correlates early and the feature/response aspect late in the post-stimulus phase (Fig. 2B). In addition, Fig. 2C shows the scatterplots for the correlations at each task aspects’ peak decoding accuracy (see Fig. 1C). It is noteworthy that just as for the trial-to-trial relationships, the individual-differences relationships appear to express feature-specific effects on performance, rather than an unspecific, noise/decodability factor. With its small sample size, the current experiment was not originally designed to examine individual differences. Therefore, these analyses need to be treated as exploratory and require replication. Nevertheless, confidence in the results is strengthened by the fact that the relationships are strikingly robust and are, both in terms of involved features and their time course, highly consistent with the within-individual relationships.

### Relationship between representations

The notion that cues activate task-level control settings, which in turn bias stimulus and eventually response representations, leads to a straightforward prediction about the sequence in which different lower-level representations should be related to the strength of attentional settings. Figure 3 presents for each timepoint the relationships between the classifier confidence for task-level information and each of the other aspects (to avoid clutter, we omit the distractor here, for which the relationship was close to zero throughout). As expected, early in the prestimulus phase, the strength of attentional sets was coupled with the strength of cue representation, likely indicating the activation of the attentional sets based on the cue^5^. Following stimulus onset, a correlation with the target location emerged and subsequently, a correlation with the response information. This pattern is again consistent with the parallel-activation account, where attentional sets coordinate lower-level representations in a concurrent manner (see Fig. 1B).

### Effects of task switching

In the results presented so far, we had ignored potential effects of trial-to-trial changes in tasks—typically of major interest in task-switching research^1^. In fact, our version of the task-switching paradigm was optimized towards EEG decoding analyses, not towards producing large switch effects (i.e., both the use of long inter-trial intervals and of spatially separate, task-related features can be expected to reduce between-task competition). Indeed, behavioral switch costs were small (see Table 1). Nevertheless, we examined the degree to which the switch/repeat contrast plays out in the decoding results. We constrained our analyses a-priori to the 150 ms intervals centered around the peak of the activation trajectories for each feature (see Fig. 1C). In addition, given the strong relationship between RTs and decoding accuracy for task, stimulus locations, and response, we also conducted a median split into fast and slow RT trials. The median-split was conducted within each subject, task, and switch condition; values were than averaged across tasks and subjects, but presented separately for no-switch and switch trials. The dominant aspect in Fig. 4 is again the strong relationship between RTs on the one hand and task, target location, and response representations on the other. In addition, switching tasks led to weakened task-set representations, both in general (switch main effect: *F*(1,19) = 6.69, *p* = 0.018), but in particular on slow-RT trials (fast/slow × switch interaction: *F*(1,19) = 6.62, *p* = 0.019). Also, distractor representations were increased on switch trials, *F*(1,19) = 5.23, *p* = 0.034. Thus, at the time of peak attentional-set activation, decoding of task-level information was less robust on switch than on no-switch trials, whereas information related to the competing task was more strongly expressed.

### How abstract are attentional sets?

In our paradigm, attentional settings are confounded with attention to different visual features (i.e., color versus orientation). Thus, the results reported so far do not allow strong conclusions about the question to what degree the task-level decoding reflects representations of abstract task rules, or the engagement of task-correlated sensory or response-related features. In fact, as shown in the Supplemental Material, there were marked between-task differences in the time course of encoding target locations, with color decoding being stronger and earlier than orientation decoding. Arguably, even if task-level decoding is driven by such aspects they would still be the consequence of top-down, task-level representations and thus indirectly reflect the strength of top-down control through such abstract representations. However, we can also conduct additional analyses to explore the abstractness of the information decoded on the level of tasks.

As a first step, we analyzed how task-set decoding generalizes across critical stimulus/response aspects. For this purpose, we split the data into four target positions by two features/responses (=8) bins. We then trained classifiers to discriminate task for each of these bins and tested for generalization both with left-out trials within the source bin and for the remaining seven bins. The insert in Fig. 1C presents the time-course of average, within-bin classification accuracy and across-bin classification accuracy (for each source bin, averaged across all seven generalization bins). As evident, accuracy for generalization analyses was somewhat reduced, but remained very robust. This result confirms that at least a substantial part of the decodable task-related information is indeed of a relatively abstract nature.

In a second step, we can ask to what degree the predictive power of task-level information (see Fig. 2A) is associated with abstract information versus task-correlated, lower-level aspects. Thus, instead of task-set classifier confidence, we used the degree of generalization (see insert to Fig. 1C) to predict RTs. As shown in the insert to Fig. 2A, the generalizable aspect of the decoded task-level representation remained a robust predictor of performance. In the Supplemental Material, we report an additional analysis, where we controlled in the predictive analyses (Fig. 2A) for the degree to which lower-level representations generalized across tasks on a trial-by-trial basis (see Fig. S3). Again, we found no change to the overall predictive pattern, confirming that the decoded task-set representations were relatively abstract (Fig. 4A).

### Consistency of task decoding patterns

We used an individual-specific decoding approach because we had no a-priori predictions about the frequency bands and electrode locations that capture task-specific information. Nevertheless, it would be useful to know to what degree such an individual-specific approach is in fact warranted. Therefore, we computed for each subject, electrode, and frequency band the power across the 150 ms around the average, peak decoding accuracy for task sets (see Fig. 1C) and compared these across the two tasks. We then correlated the resulting vector of 80 (20 electrode × 4 frequency bands) *t*-values for the task contrast for each participant with that of every other participant. Figure 5 shows the histogram of the resulting correlations; the average is *r* = 0.13 (computed by averaging z-transformed correlations and re-transforming the mean into an *r* coefficient) and the range, although tilted towards the positive direction is very large. This result suggests that the pattern separating the two tasks is fairly idiosyncratic and therefore justifies an individual-specific decoding approach.

## Discussion

When people need to respond to a given stimulus in a flexible, context-dependent manner, the flow of information processing cannot rely on sensory or response representations alone. Rather, stimulus and response selection processes have to be constrained by representations of the current context, goals, and/or stimulus-response rules. There is a substantial literature using behavioral^1^ and neuroimaging methods^37–40^ on how we select and change representations that enact top-down control. However, compared to the recent progress made using neurophysiological methods in primates (Siegel *et al*.^17^), it has been much more difficult to precisely characterize the place and the relevance of such representations within the overall information processing cascade.

By decoding information about all potentially relevant features from EEG signals, we revealed a plausible sequence of active representations of target and distractor locations, as well as of response choices. For example, the timing of target/distractor location representations was highly consistent with recent work using eye-tracking to assess the dynamics of attentional allocation to task-relevant and irrelevant features^12,26^. More importantly, our results also reveal the time course for both cue and attentional-set representations. Task cues were highly decodable as soon as the cue was presented during the prestimulus phase, but were less strongly expressed once the stimulus appeared. In contrast, task representations exhibited the reverse pattern: Their activity ramped up only slowly during the post-cue/pre-stimulus phase, but showed a very strong presence during the entire stimulus-to-response.

The pattern of average, cue and attentional-set activation trajectories is of some theoretical interest by itself. For example, contrary to one prominent model^4^, the fact that task-level decoding accuracy is much higher than cue decoding accuracy (at least after stimulus presentation) suggests that task-set activity is more important than superficial cue information in controlling lower-level representations. Yet, average decoding accuracy allows no firm conclusions about the functional relevance of the different representations. This is where decoding scores on the single-trial level yield important, additional information. Using these scores to predict trial-to-trial variability in RTs, allowed us to determine the representations that drive performance in a time-resolved manner (Fig. 2). Interestingly, the pattern of predictive relationships indicates that cue representations do not explain variability in performance. In contrast, task representations emerged as a very robust predictor of trial-to-trial variability in RTs. As for the trajectories of decoding accuracy (see Fig. 1C), the explanatory power of task-level representations was again largely limited to the post-stimulus phase and was most robust when also stimulus and response effects are particularly strong. Task-level information (but not cue information) also emerged as a robust predictor of inter-individual differences in performance—a result that will need to be confirmed with larger samples. In addition, an analysis of interrelationships between task and lower-level decoding scores (see Fig. 3) indicates that attentional sets were coupled in the post-stimulus phase initially with the target-location representations, and thereafter also with feature/response representations. Combined, these results strongly suggest that lower-level representations are configured through more abstract task or attentional settings, rather than through superficial cue representations. Further, the trajectory of average representational strength and the predictive pattern is most consistent with the parallel activation model (Fig. 1B), where attentional sets can shape lower-level processes in a concurrent manner^3^.

Our results do not rule out the possibility of functionally relevant, preparatory activity before stimulus/response processing sets in. In fact, the use of cue information to retrieve the current attentional set is a necessary process that clearly happens within the cue-to-stimulus interval^5^. The early inter-relationship between strength of cue and task representations likely is an expression of this retrieval activity (Fig. 3). It is reasonable to assume that the 300 ms interval between cue and stimulus interval was sufficient to absorb major, within-individual or between-individual variability in the duration or quality of this process, thus preventing any predictive effects of prestimulus cue or task-set representations from revealing themselves. Also, it is very well possible that with longer preparatory intervals, greater proactive task-set activity might be observed. Both behaviorally and in EEG or fMRI neuroimaging studies, preparation effects are well documented^27,41–44^. However, the fact that task-set representations were strongest, and also most predictive of performance in the presence of stimulus and response representations suggests that a key function of attentional sets is to regulate these lower-level representations in a concurrent manner. This conclusion is also consistent with a large body of behavioral work suggesting that task-selection costs cannot be easily eliminated through opportunity for preparation^1^.

The most important limitation of the current work is that we cannot be certain that the decoded, task-level information reflects exclusively abstract rule-type representations. As the two different tasks required attention to different visual aspects and different S-R rules it is possible that between-task decoding is driven to a large part by these aspects (see Supplemental Material). Even if that was the case though, given that the bottom-up stimulation did not differ across tasks, these aspects must reflect the degree of task-related engagement, which at the very least is an indirect reflection of higher-level control representations.

We also report several results suggesting that the task decoding accuracy we observed does in fact represent the strength of relatively abstract, task or attentional settings. For example, in the predictive analyses, task-level classifier confidence explained substantial, within-individual variability in RTs over and above the predictive relationships between RTs and lower-level aspects. We also demonstrated that task representations generalized across target positions and features/responses. Furthermore, these generalization scores proved nearly as predictive of within-individual and between-individual variability in performance as the regular decoding accuracy.

Because we had no strong a-priori predictions about frequency bands or electrode sites that might carry the relevant information, we used a strict bottom-up approach to decode tasks/attentional sets. Obviously, decoding analyses could be restricted to specific frequency bands (or electrodes) to test predictions about which parts of the signal contain theoretically relevant information^45^. Frequency-specific decoding analyses would also address another limitation of the current approach. Time-frequency analysis comes with some degree of blurring of the precise temporal characteristics—which for example is the likely reason for the above-chance increase of decoding accuracy for the target feature before stimulus onset (see Fig. 1C). Given that the degree of blurring is larger for lower than higher frequency bands and that there is no control over the contribution of each frequency band to the overall decoding accuracy, it is difficult to determine the degree to which temporal precision is compromised. When precise temporal relationships among features need to be assessed (e.g., which feature is activated first) then it would be useful to conduct decoding analyses in a frequency-specific manner. In the current case, however, none of our conclusions rest on precise, temporal comparisons.

In key aspects, our pattern of results was remarkably similar to earlier-mentioned results reported by Siegel *et al*.^17^. In particular, cue information was highly prominent during the pre-stimulus phase, but then tapered off in the post-stimulus phase. Task information emerged concurrently with cue information, but then increased dramatically as stimulus and response choice information was processed during the stimulus phase. The convergence of results across species and methods suggests that some of the same information that is conveyed through neuron-level recordings can also be extracted through scalp-level EEG signals. The fact that we were able to extract information about task-relevant features through relatively sparse recordings from the scalp is generally consistent with the fact that in Siegel *et al*., both higher-level and lower-level aspects were represented throughout all cortical regions, albeit with varying strengths across regions.

To summarize, we show here that EEG-based, trial-by-trial decoding analyses can clarify the relative role and the temporal dynamics of both lower-level stimulus/response, as well as more abstract attentional-set representations. In particular, the ability to pinpoint the exact source of performance differences within a cascade of simultaneously evolving representations is a unique feature of this approach.

## Supplementary information

Supplementary Material
