# Author Correction: EEG Decoding Reveals the Strength and Temporal Dynamics of Goal-Relevant Representations

**DOI:** 10.1038/s41598-020-65539-3

**Published:** 2020-05-13

**Authors:** Jason Hubbard, Atsushi Kikumoto, Ulrich Mayr

**Affiliations:** University of Oregon, Eugene, OR 97403 United States

Correction to: *Scientific Reports* 10.1038/s41598-019-45333-6, published online 21 June 2019

In Figure 2, the correct panel B is missing and panel C is incorrectly given as panel B. The correct Figure 2 appears below as Figure 1.

**Figure 1:**
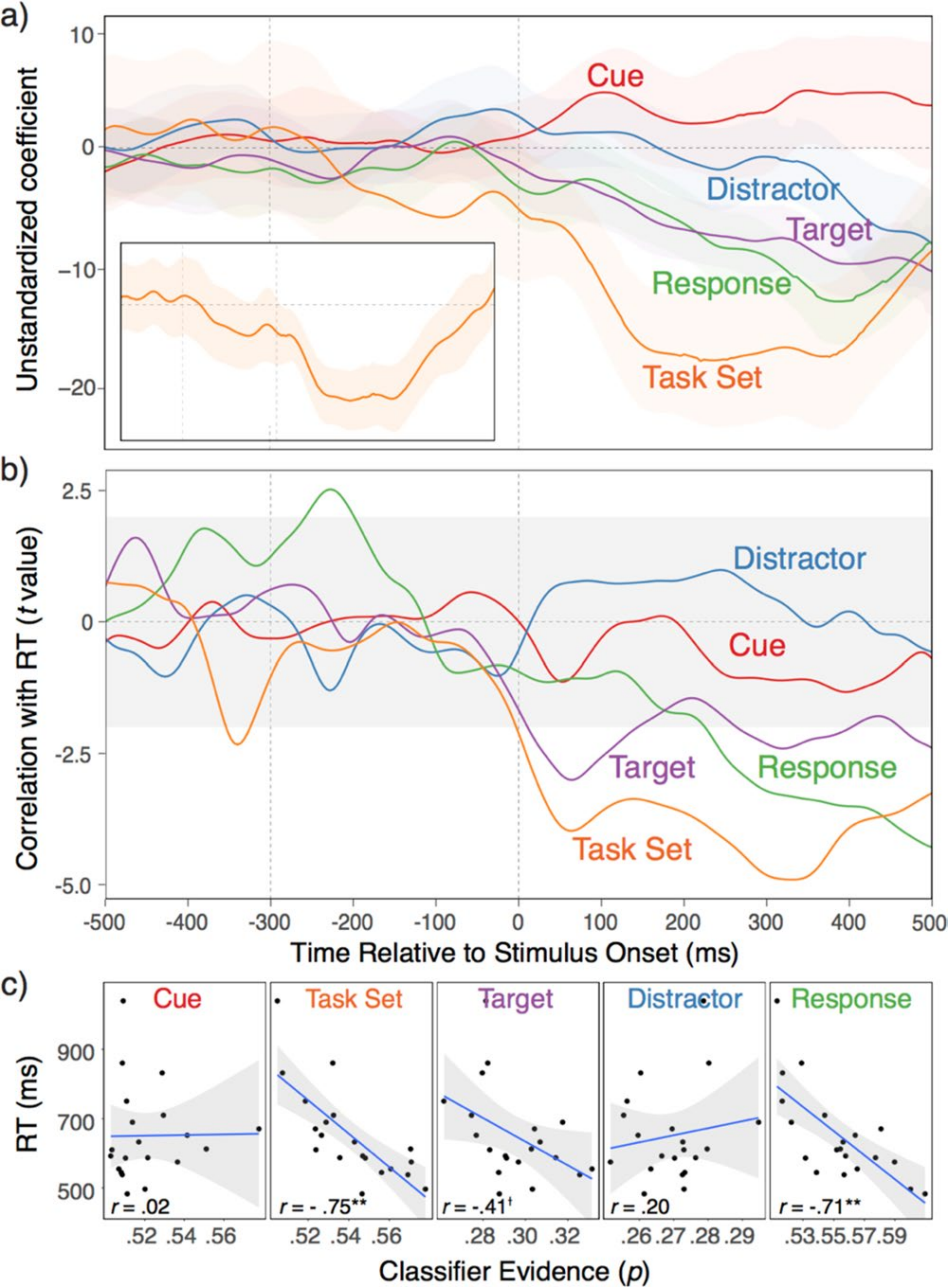
.

