# Supplementary material for: EEG Decoding Reveals the Strength and Temporal Dynamics of Goal-Relevant Representations
Source: Sci Rep. 2019 Jun 21;9:9051. doi: 10.1038/s41598-019-45333-6 (PMC6588723; doi:10.1038/s41598-019-45333-6)
Supplement: Supplementary file 1 — Supplementary Material [file 41598_2019_45333_MOESM1_ESM.pdf]

EEG Decoding Reveals the Strength and Temporal Dynamics of Goal-Relevant Representations

Jason Hubbard, Atsushi Kikumoto, and Ulrich Mayr

University of Oregon

Supplementary Material

Ruling out Eye-Movement Artefacts

Even though we excluded trials on which the EOG indicated eye movements, it is difficult to rule out that subtle, remaining eye movements might contribute to task-decoding results. In order to provide additional safeguards in this regard, we conducted a follow-up analysis, in which we grouped all trials into those with large and those with a small, absolute, horizontal EOG signals (using a within-subject median split; note that all eye-movements were still below the exclusion threshold). We then decoded the task feature separately in the same manner as in the main analyses, within each of these trial types. The reasoning behind this analysis is that should eye movements contribute to task decoding results then decoding accuracy should be substantially better on trials with larger than on trials with smaller eye-movements (Foster et al 2015).

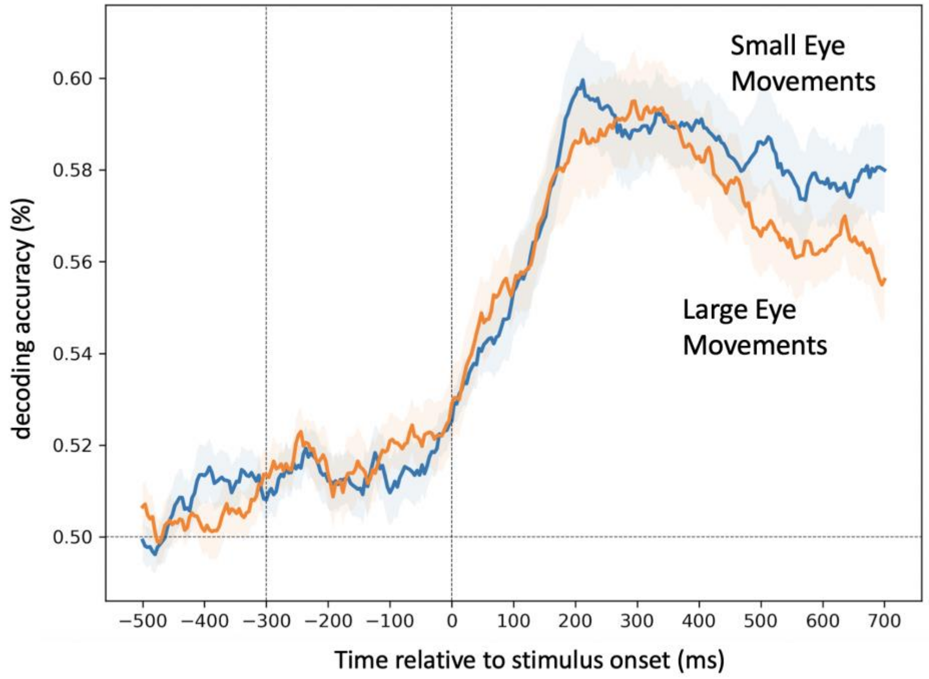

***Figure S1**.* Task decoding accuracy for small and large eye movements.

Figure S1 shows the decoding accuracy for both categories of trials. As apparent, there was very little difference between the decoding accuracy for large and for small eye-movement

trials. This results strongly suggests that at least for the decoding of the theoretically, most important feature, namely the task, eye movements were not a relevant factor.

Task-Specific Effects

In Figure 1c in the manuscript we average decoding results across tasks. However, it is likely that the different stimulus dimensions (color vs. orientation) for the two different tasks differ in their attentional demands. Therefore, we also present the decoding results here separately for the color and the orientation task (see Figure S2).

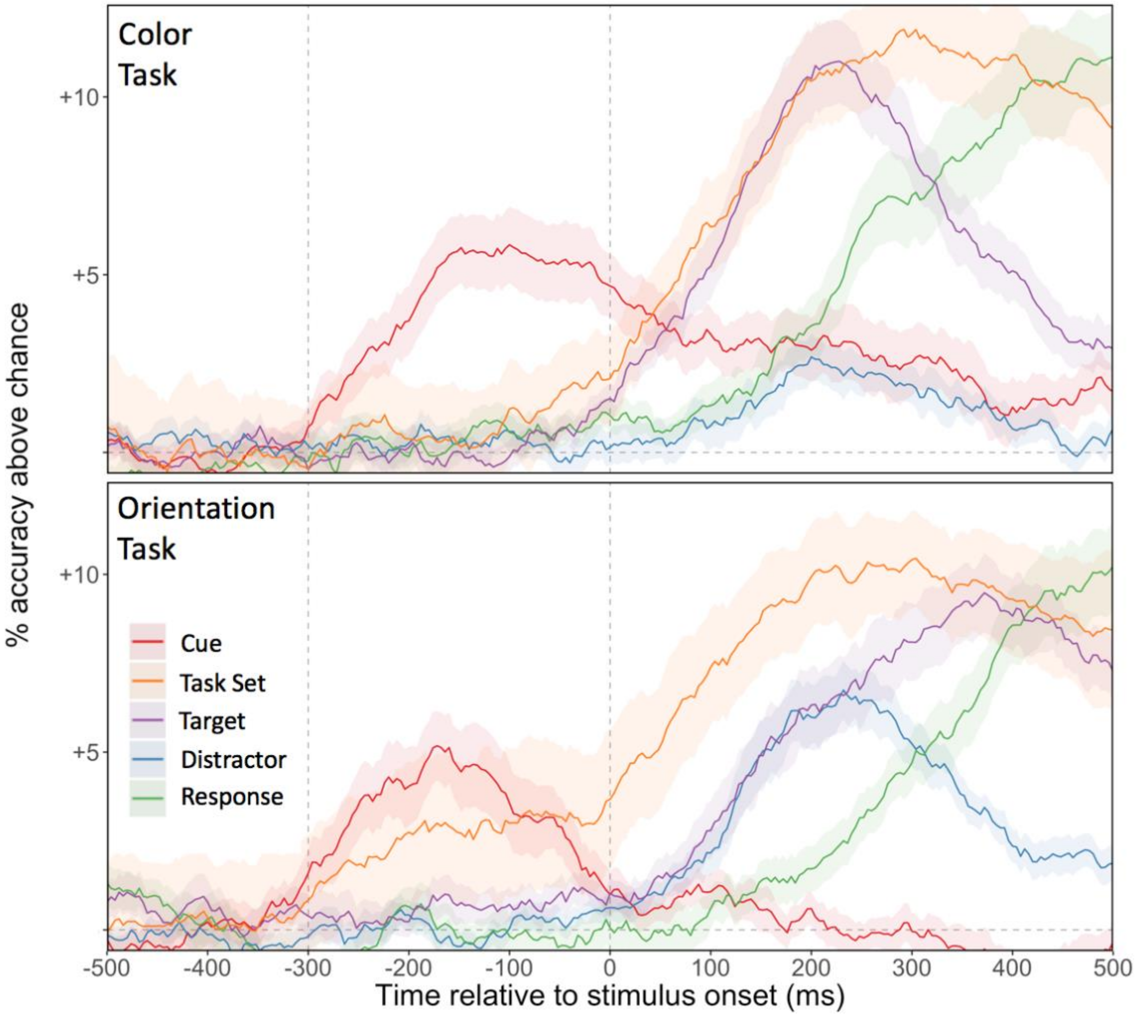

Figure S2. Decoding accuracy for the color and the orientation task.

As obvious, the color target is decoded with much higher accuracy than the orientation distractor immediately after stimulus onset. By contrast, the orientation target and the color distractor are initially equally well represented and are differentiated only around 250 ms. This overall pattern matches up remarkably well with so far unpublished results from task-switching experiments using eye-tracking, where we find qualitatively identical results for the probability of fixations to color targets and distractors compared to shape/orientation targets and distractors (e.g., Kikumoto, Hubbard, & Mayr, 2016; Mayr, Kuhn, & Rieter, 2013). Thus, these task-specific differences provide additional validation for the decoding accuracy results. At the same time however, such differences between tasks need to be considered in interpreting the decoding of the task in terms of representations of attentional sets. It is possible that task decoding reflects at least in part such task differences in lower-level features.

To document and quantify such task differences, we can examine the generalization of trained classifiers across tasks. For each time point, we performed cross-classifications where we trained a classifier to distinguish target/distractor locations and responses for one task and then tested the classifier both on left-out trials for the same task, and for the alternative task. For target and distractor locations, we find indeed that compared to within-task decoding, across-task generalization is relatively weak for target locations and essentially absent for distractor representations (Figure S3). Likely, this difference can be attributed to the different time-courses for the decoding accuracy of target/distractor locations across the two tasks. For responses, the decoding accuracy time course is more similar across tasks (see Figure S1) and also the difference between within-task and between-task classification accuracy is much smaller than for locations.

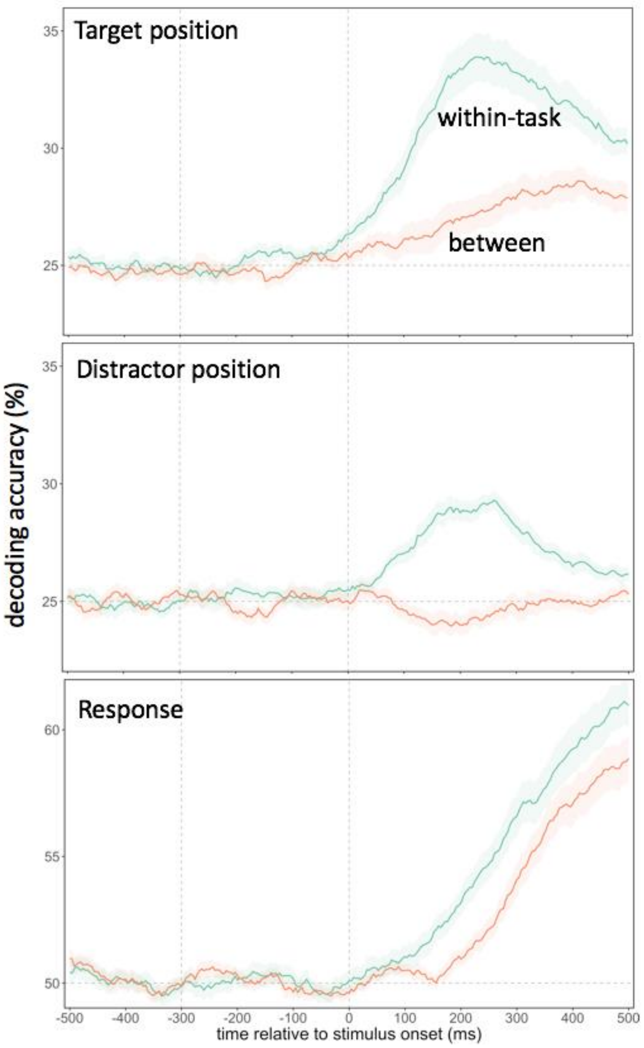

Figure S3. Generalization of decoding of the target position, distractor position, and response across tasks.

In order to examine the degree to which the decoding of tasks relies on such lower-level differences, we analyzed how task-set decoding generalizes across critical stimulus/response aspects. Specifically, we split the data into four target positions by two features/responses (=8) bins. We then trained classifiers to discriminate task for each of these bins and tested for generalization both with left-out trials within the source bin and for the remaining seven bins. The insert in Figure 1c presents the time-course of average, within-bin classification accuracy and across-bin classification accuracy (for each source bin, averaged across all seven generalization bins). As evident, accuracy for generalization analyses was somewhat reduced,

but remained very robust. This indicates that a substantial share of the decoding accuracy can be attributed to the abstract attentional-set representation, rather than to specific stimulus aspects.

Given that distractor representations generally were relatively weak we focused in the main manuscript for these analyses on target representations (dividing up the data by target and distractor positions simultaneously would have yielded bin sizes that are too small for decoding analyses). However, we repeated the above analysis, using the combination of distractor positions and feature/responses and obtained if anything greater degree of generalization.

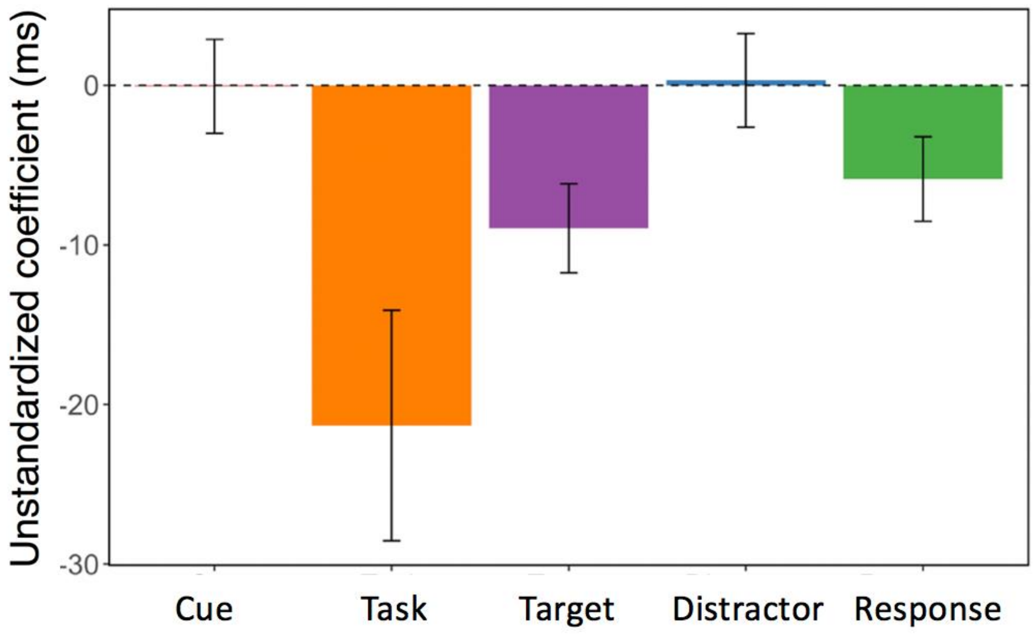

Figure S4. Within-individual relationships between each task aspect's decodability during the period of its peak decoding accuracy (see Figure 1c) and RTs. Coefficients are from a multilevel, linear model with all task aspects entered simultaneously.

Trial-Level Relationships

For predictive relationships presented in Figure 2a, all aspects were presented simultaneously for *each* timepoint. This analysis might not do adequate justice to potential asynchronous relationships between the different aspects and RTs. Therefore, we identified the time point with the maximum decoding accuracy for each aspect (see Figure 1c) and averaged

the (logit-transformed) decoding accuracy across a 150 ms window centered around that point. For the response aspect, the maximum decoding accuracy was towards the end of the 500 ms interval, so we simply averaged the period from 350-500 ms. We then entered these scores as predictors of RTs in the same type of a mixed-effects model used for the timepoint-by-timepoint analyses. Again, the attentional-set emerges as a particularly strong predictor of RT variability, over and above the predictive relationships with the target and the response representation (see Figure S4).

In order to ensure that the predictive relationship found for task-level decoding (as shown in Figure 2a) is in fact due to abstract attentional-set representations, we conducted two sets of control analyses. First, we repeated the just-mentioned predictive analyses, but replaced the task predictor with the averaged generalization score from the analyses reported in the insert in Figure 1. The logic here is that the degree of generalization serves as a (inverse) measure of lower-level between-task differences. Thus, the degree to which the predictive power of task-level representations results from between-task differences in lower-level representations, the predictive effects should be attenuated by controlling for trial-by-trial information about these between-task differences. The insert in Figure 2a shows that the predictive pattern for task remains virtually unchanged when using these generalization scores.

The second analysis follows the same general logic. However, here we calculated for each time point the difference between within-task and between-task generalization of target, distractor, and feature/response decoding (see Figure S3) as indicators of task-specificity of the lower-level representations. We then included these three task-specificity scores as additional predictors for the analyses presented in Figure 2a. Again, if the predictive power of the task-set representation is dependent on trial-to-trial variations in the task-specificity of lower-level aspects then including the task-specificity scores should reduce or eliminate the predictive relationship between task-sets and RTs. As shown in Figure S5, results indicated a robust predictive relationship between task-level decoding accuracy and RTs. These results reinforce

the conclusion that it is the representational strength of a relatively abstract, attentional set that carries the predictive power.

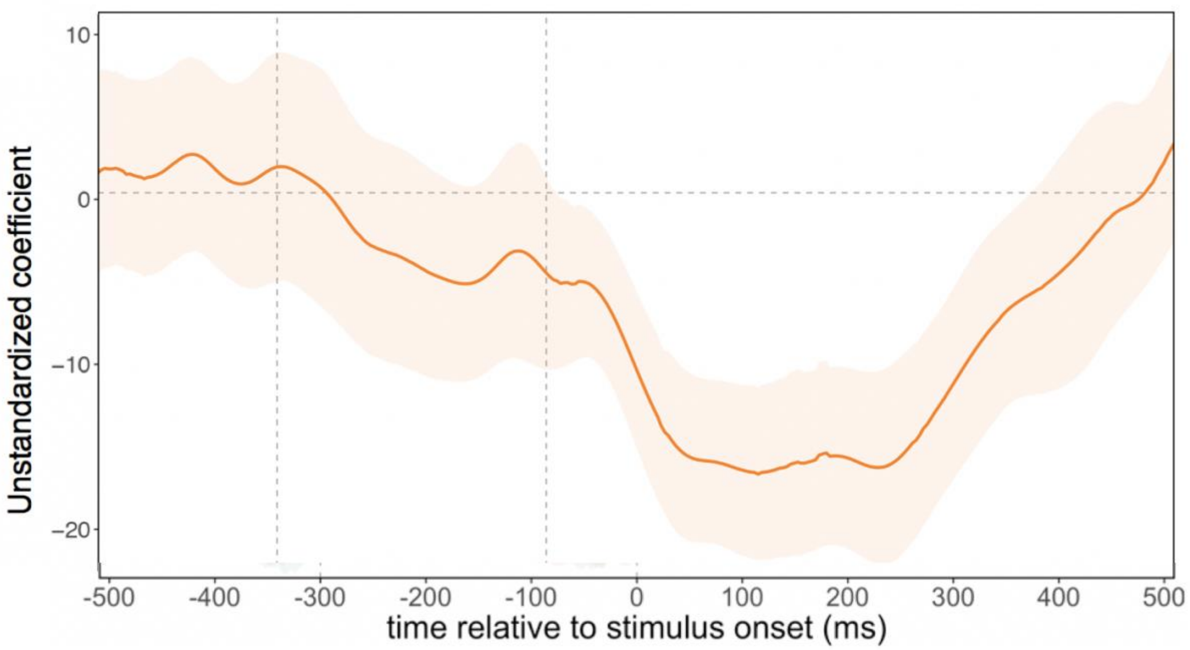

Figure S5. Coefficient for the task-level decoding as a predictor of trial-by-trial variability in RTs while controlling for the difference between within-task and between-task generalization of target, distractor, and feature/response decoding (see Figure 2S).

References

Buschman, T. J., Denovellis, E. L., Diogo, C., Bullock, D., & Miller, E. K. (2012). Synchronous oscillatory neural ensembles for rules in the prefrontal cortex. *Neuron*, *76*, 838-846.

Cohen, M. X. (2014). *Analyzing neural time series data: theory and practice*. MIT Press.

Delorme, A., & Makeig, S. (2004). EEGLAB: an open source toolbox for analysis of single-trial EEG dynamics including independent component analysis. *Journal of Neuroscience Methods*, *134*, 9-21.

Foster, J. J., Sutterer, D. W., Serences, J. T., Vogel, E. K., & Awh, E. (2015). The topography of alpha-band activity tracks the content of spatial working memory. *Journal of Neurophysiology*, *115*, 168-177.

Fries, P. (2005). A mechanism for cognitive dynamics: neuronal communication through neuronal coherence. *Trends in Cognitive Sciences*, *9*(10), 474-480.

Kikumoto, A., Hubbard, J., & Mayr, U. (2016). Dynamics of task-set carry-over: evidence from eye-movement analyses. *Psychonomic Bulletin & Review*, *23*, 899.

Mayr, U. (2001). Age differences in the selection of mental sets: the role of inhibition, stimulus ambiguity, and response-set overlap. *Psychology and Aging*, *16*, 96.

Mayr, U., Kuhns, D., & Rieter, M. (2013). Eye movements reveal dynamics of task control. *Journal of Experimental Psychology: General*, *142*(2), 489.

Monsell, S., & Mizon, G. A. (2006). Can the task-cuing paradigm measure an endogenous task-set reconfiguration process? *Journal of Experimental Psychology: Human Perception and Performance*, *32*, 493.

Pedregosa, F., Varoquaux, G., Gramfort, A., Michel, V., Thirion, B., Grisel, O., ... & Vanderplas, J. (2011). Scikit-learn: Machine learning in Python. *Journal of Machine Learning Research*, *12*, 2825-2830.
